# Effects of saccade delay, side of deficit, and training on detection of catch-up saccades during head-impulse test in virtual-reality-enhanced mannequin

**DOI:** 10.1038/s41598-023-29801-8

**Published:** 2023-02-15

**Authors:** Ambre Charlery-Adèle, Caroline Guigou, Julien Ryard, Mathis Chartier, Michel Toupet, Christophe Guillet, Férédric Mérienne, Alexis Bozorg Grayeli

**Affiliations:** 1 Otolaryngology Department, Dijon University Hospital, Dijon, France; 2 ImVia, Laboratory of Imagery and Artificial Vision, EA 7535, Burgundy University, Dijon, France; 3 Arts et Métiers Institute of Technology, LISPEN, HESAM Université, 71100 Chalon-Sur-Saône, France; 4 Arts et Métiers Institute of Technology, LiSPEN EA 7515, Burgundy University, 71100 Chalon-sur-Saône, France

**Keywords:** Auditory system, Oculomotor system, Sensory processing, Experimental models of disease

## Abstract

In this study, a training simulator for the examination of dizzy patients based on a virtual-reality-enhanced mannequin (VREM) was developed to evaluate the detection of catch-up saccades during head impulse test (HIT) and the effect of training in VREM. For novices (n = 35), 2 trials were conducted before and after a training session. Experts (n = 7) were submitted to an evaluation session. In each trial, a left or a right horizontal canal deficit with an overt catch-up saccade (delay between 110 and 320 ms) was randomly presented. Participants scored the difficulty in performing the maneuver, in recognizing the saccades, and the self-confidence in the diagnosis using a visual analogue scale (VAS). Saccade delay significantly influenced the performance. Training significantly improved the sensitivity in the residents (69.1% before to 97.9% after the training, p < 0.001, Fisher's exact test, n = 560 tests), surpassing experts’ performances (p < 0.001, versus 87% in experts, Fisher's exact test). The specificity also increased to the expert level (78% before to 95% after the training, and 95% in experts, p < 0.001, Fisher’s exact test). The VAS showed a decrease difficulty to execute the HIT, with an increase in the confidence after training. VREM improved the HIT execution performance and the confidence in novice practitioners.

## Introduction

The examination of a dizzy patient requires training in observational and motor skills^1^. The unilateral paresis of a semicircular canal (SCC) can be diagnosed by a head impulse test (HIT) as a rapid and reproducible clinical tool^2–4^. Several studies agree that HIT combined with 2 other clinical tests (nystagmus and skew) (HINTS) could distinguish acute peripheral vertigo from central vertigo with a sensitivity of 100% and a specificity of 96%, which would be superior to the performance of early MRI^3,5–9^. A more recent publication reported that HINTS performances were higher when performed by trained neurologists in comparison to tests conducted by emergency physicians: 96.7% of sensitivity and 94.8% of specificity versus 83% and 44%, respectively. This observation highlights the importance of the training^10^.

The rational of the HIT is to evaluate the lateral SCC function through the vestibulo-ocular reflex (VOR). The patient should be seated, head straight. The examiner faces the patient, places his/her hands on either side of the head, and asks him/her to fixate a visual target in front of him/her^11,12^. For lateral SCC, a small (10°–20°) but rapid rotation is applied to the patient’s head (speed > 100°/s)^13–16^. The conjugated action of lateral SCCs and oculomotor system contribute to the VOR and maintain the gaze on the target. In case of a vestibular deficit, during the rotation to the side of the deficit, the eye leaves the target and drifts to the side of the impulse. The retinal image slip triggers a catch-up saccade toward the target after a variable delay ranging 150–200 ms for those visible to the examiner^2,17^. Although the drift cannot be detected by the physician’s eyes, the visible catch-up saccade indicates an SCC weakness on the side of the impulse^2^. Evidently, the detectability of these saccades depends on their latency after the impulse. Video-head impulse tests (vHIT) show a relatively large range of saccade delays: those occurring after the head movement are called “overt saccades”, while those detected during the impulse are designated as “covert saccades”^12,14,18,19^. For anterior and posterior SCCs, the test is more complicated since the impulse should be in the left-anterior right-posterior (LARP) or right-anterior left-posterior (RALP) planes^18,20^. In these planes, the head movements can be limited, less reproducible and the patients’ eyes less accessible to examination^2,11^.

This test remains difficult in current clinical practice since 34% of the deficits are only manifested by covert saccades, undetectable without video recording^14^. Studies comparing the HIT diagnostic performance to the caloric tests show that the HIT sensitivity is even less than the rate expected after the deduction of the covert saccades: it culminates at only 34–39%. However, it provides a good specificity, ranging from 95 to 100%^18,21,22^. Low detection of the overt saccades in clinical studies raises the questions of its detectability by the examiner when the latencies are short (< 150 ms) and the necessity of training. The amplitude of the saccade could also play a role in the detection of the catch-up saccade (higher probability of detection with increasing amplitude)^23^.

The correct execution and interpretation of HIT is complex since it involves both a correct head movement and observational capacities^2,5,24^. The speed of the head impulse is directly related to the amplitude of the saccade and therefore influences its visibility^12^. Consequently, HIT cannot be correctly executed without training^4^. Clinical experience is difficult to muster, since for practical reasons, the test cannot be repeated several times by different physicians and trainees on the same patient, and the test results cannot be confronted to the ground truth immediately. A previous work has been reported on HIT training for novices using a vHIT device. The training focused only on the head impulse execution (i.e. number of HITs accepted, speed of head rotation) and did not concern the detection of catch-up saccades or the factors that could influence it^25^.

To our knowledge, there is no training model or simulator for this test and a virtual-reality-enhanced mannequin (VREM) providing haptic feedback appears to be the most appropriate system for this type of training.

The aim of this project was to evaluate a VREM for HIT training, assess the detection rate of catch-up saccades as a function of their latency, and analyze the effect of anterior experience, training, and the side of the deficit on their detection.

## Results

Regardless of the delay, the catch-up saccades were detected in 69.1% of cases (true positive rate or sensitivity, n = 560 tests) by the residents before training, and in 86.6% by the experts (n = 112 tests). Sensitivity (or true positive rate) was defined by to the ability to identify cases catch-up saccades on the correct side in case of a unilateral deficit.

Training significantly improved the sensitivity in the residents to 97.9% (p < 0.001, versus before training, Fisher's exact test), surpassing experts’ performances (p < 0.001, versus 87% in experts, Fisher's exact test, Table 1).

**Table 1:** Sensitivity and specificity of the HIT test.

	Novices	Trained novices	Experts
Global			
Sensitivity	0.69	0.98	0.87
Specificity	0.78	0.95***	0.95
Right deficits			
Sensitivity	0.70	0.97	0.88
Specificity	0.82	0.97	0.96
Left deficits			
Sensitivity	0.69	0.99	0.86
Specificity	0.73*	0.93*	0.93*

Performances evaluated on 560 tests for novices before and after training and on 112 tests for experts. Each side represented 260 tests for novices and 56 for experts. *p < 0.05 versus the right side, Fisher’s exact test. ***p < 0.0001 versus novices, Fisher’s exact test.

**Figure 1:**
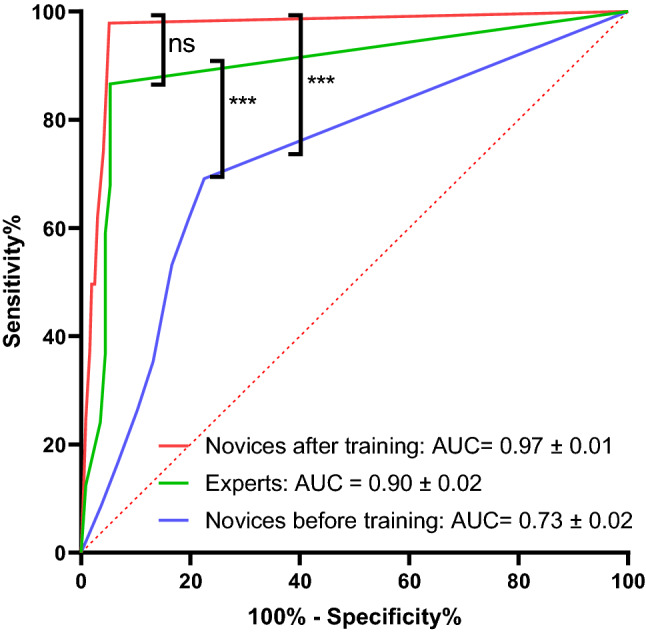
ROC curves in experts and in residents before and after training. ***p < 0.001, and *ns* not significant.

The specificity (true negative rate) corresponded to the ability of the test to correctly identify cases with no deficit and no catch-up saccades. The overall specificity of the test in the novice group increased from 78% before to 95% after the training (p < 0.001, Fisher’s exact test, n = 560 tests) reaching the expert level (95%). ROC analysis of expert and novice performances confirmed these observations by showing a significant AUC increase after training in novices (Fig. 1).

**Figure 2:**
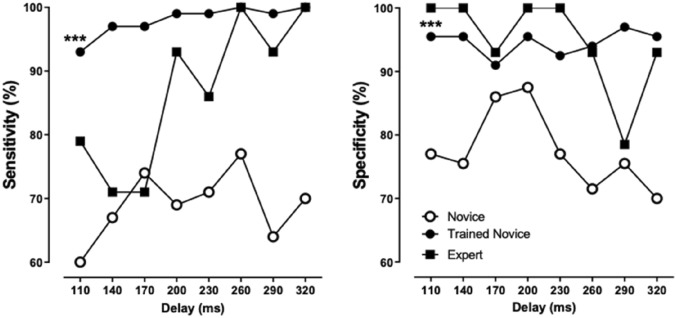
Sensitivity and specificity as a function of saccade delay. ***p < 0.001 for novices before versus after training at all delays (n = 70 for each delay, Fisher's exact test), ^£^p < 0.05 experts versus novices after training (n = 70 for novices and n = 14 for experts, Fisher's exact test). *p < 0.05 and ***p < 0.001 for the 110 ms delay versus delays > 230 ms in the same group (for each delay, n = 70 for novices and n = 14 for experts, Fisher's exact test).

The saccade delay appeared to influence the sensitivity in both groups: saccades occurring at 110 ms were less often detected than those occurring beyond 230 ms (Fig. 2).

In experts, a cut-off value around 200 ms could be observed below which the sensitivity decreased abruptly. The effect of training was most prominent for short delays, with novices surpassing experts in terms of sensitivity at 140 and 170 ms. Specificity also increased after training in novices to match the expert performances (Table 1) especially at upper and lower limits of the tested saccade delays (Fig. 2).

While the deficit side did not seem to influence the sensitivity, it affected the specificity in novices with a lower value in left deficits than in right weaknesses before and after training (Table 1). This difference was not significant for the experts (Table 1). The examiner’s hand laterality did not influence the performances significantly. By comparing the performances in all 3 left-handers (2 novices and 1 expert) to 39 right-handers (33 novices and 6 experts) after the training, there was a trend for higher sensitivity in left-handers (Table 2).

**Table 2:** Effect of body laterality on HIT performances.

	Sensitivity (%)		p value	Specificity (%)		p value
	Right-handed (n = 312 tests)	Left-handed (n = 24 tests)		Right-handed (n = 312 tests)	Left-handed (n = 24 tests)	
Right deficit	97.1	100	1.0	95.2	95.8	1.0
Left deficit	92.0	100	0.23	96.2	100	1.0
Total	100	97.9	0.16	94.6	95.7	0.71

Left-handed group comprised 2 novices and one expert and the right-handed group included 33 novices and 6 experts. For the novice group, only the performances at the final test were analyzed. Comparisons between left-handed and right-handed groups were conducted by a Fisher’s exact test.

**Figure 3:**
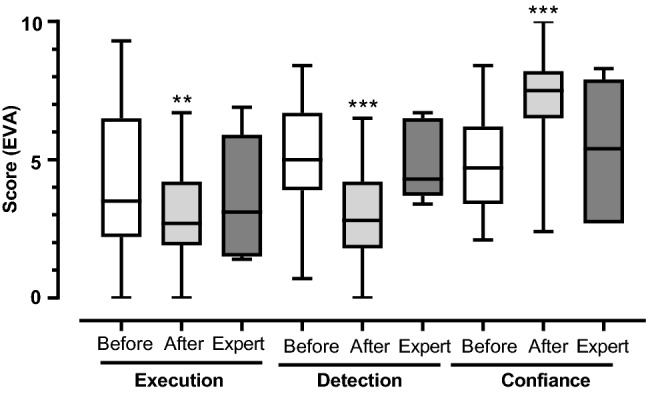
Difficulty of test execution, of saccade detection and detection confidence level estimated by visual analogue score among novices before and after training and in experts. The boxes represent the 95-percentile confidence interval, the horizontal bar the mean and the whiskers the range. **p < 0.03 and ***p < 0.001, two-way ANOVA followed by Bonferroni test.

The self-assessment regarding the difficulty to execute the maneuver and to detect the saccades showed a decrease in difficulty scores after training. Accordingly, the confidence in the correct execution and detection of the sign increased significantly (Fig. 3).

## Discussion

In this study, we demonstrated that a VREM training program based on commercially available VR tools improved the performance of the learners for HIT both in terms of sensitivity and specificity. We observed that performances were related to the saccade delays and dropped for short delays even in experts. Training improved the saccade detection especially for short delays suggesting that this tool may be interesting in training even the experts. Training led to a decrease in the subjective difficulty of performing the maneuver and detecting the sign as well as an increase in self-confidence. We also observed that simulated left deficits produced a higher proportion of false positives in this model.

Clinical studies suggest that the low sensitivity of the HIT is partly related to a short delay between the impulse and the saccade^14^. There is no relationship between this delay and the severity or the etiology of the canal deficit^14,19,26^. Saccades with shorter delays often appear several months after a vestibular deficit and are indicative of a central adaptation to the dysfunctioning VOR^27^. By reducing the retinal image slippage and stabilizing the gaze during the head rotation, a reduction of the saccade delay contributes to the improvement of the dynamic visual acuity^28,29^. Another factor related to the saccade delay is the active versus passive head rotation. The delay of the catch-up saccade can decrease because an active head rotation triggers the cervico-ocular reflex. Consequently, the eye movement is anticipated in response to the head rotation^6,27,30^. Although our training appeared to improve the detection performance, overt saccades with very short delays (< 110 ms) and covert saccades may still lead to a low sensitivity of HIT in clinical practice^21,22^. Exploring the effectiveness of the HIT at delays shorter than 110 ms and evaluating the effect of training on these very short delays will potentially enhance the performances.

The ability to resolve fine details of moving objects with a fixed head position, or static objects during the rotation of the head or body defined as the DVA is arguably a significant factor in the examiner’s performances for HIT^31^. It requires a combination of different types of eye and head movements to stabilize the image of interest on the fovea^32^. DVA is therefore related to the precision and the strategy of the examiner’s eye movements and is higher among trained athletes^33^. Experts can capture and anticipate information by processing the image features through superior indexing, organization, and storage of the information during encoding, allowing them to identify future outcomes easier^32,34^. Based on these observations, VREM can potentially increase DVA in novices by enhancing the eye–hand coordination. During the HIT, the examiner executes the head movement and expects to see the saccade. In this case, a copy of the hand motor command is sent to prepare the visual cortex (feed-forward process) potentially decreasing the detection latencies^35^. Studies in athletes suggest that this type of process can be improved by training^36^.

In this regard, analyzing the examiners eyes movement with the help of an eye tracking device integrated to the virtual reality system will potentially provide clues on the performance indicators and on eye–hand coordination aspects to improve^32,34^. Even if the expert is fixating at patient eyes, the question raises: do we look at both eyes or one eye at a time? Which eye? Do we change the examined eye through iterations etc.? We state this hypothesis as a possible question to investigate.

The side of the maneuver influenced the rate of false positives: left deficits were associated with more false positives. Was this data related to the laterality of practitioners who were mostly right-handed? To our knowledge, no other published studies corroborate these results. Our hypothesis is that the maneuver was probably less well performed on the left side in our predominantly right-handed population, resulting in distorted eye movements interpreted as saccades. The small number of left-handed subjects did not allow us to draw any conclusion on the effect of examiners’ laterality on false positives. It would be interesting in the future to study the influence of the individuals' laterality factor on the number of true or false positives.

VAS is an interesting tool for self-assessment and identification of the learning needs in trainees^37^. In similar settings, it has been demonstrated that actual competence is correlated to the self-perceived competence^38^. In our study, VAS scores suggested that training improved novices' perception of both the execution of the maneuver and the detection of the saccade.

Many students are uncomfortable in a clinical setting as it is perceived to be a stressful environment^39^. For the HIT, there is a fear of injuring the patient during the maneuver. It has already been shown that training for a clinical examination in a supervised environment increases the students' confidence^40^. Similarly, our trainees indicated a higher confidence after training. This potentially contributes to the regular use of the test in diagnostic and therapeutic decisions in daily practice. Young otologists can be trained without virtual reality equipment and only with a vHIT with very good results^25^. The interest of VREM is the possibility to increase the clinical presentations (e.g. side of deficit, delay of catch-up saccade) and to enhance observational skills. This allows the student to practice in multiple cases without the need for an external assistant. Even in the hands of a trained neurotologist, HIT will still lack sensitivity in real clinical cases because detection of covert saccades will still not be possible.

To our knowledge, this simulator is the first to realistically provide visual and haptic information for a HIT in a dizzy patient by combining a mannequin with virtual reality. The system could differentiate experts and novices in terms of sensitivity and specificity of deficit detection. After a training session in novices, we observed a significant improvement in performances reaching those of experts. The delay of the catch-up saccades influenced the detection sensitivity in both experts and novices. The subjective difficulty maneuver execution and detection as well as the level of confidence in the diagnosis also improved in novices after training to reach the level of experts.

## Materials and methods

### Simulation system

**Figure 4:**
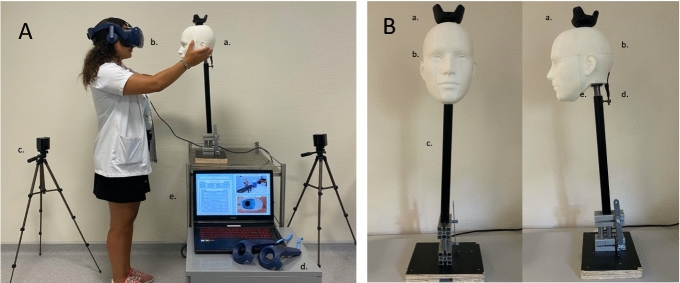
The simulation system. (**A**) The system (A) is composed of an articulated mannequin (a), a virtual reality headset (b), tracking cameras (c) to detect the mannequin and the trainee position, controllers (d) for calibration and examination purposes (not used in this study), and the laptop PC (e), providing the control interface to the trainer. (**B**) A tracker (a) is fixed on a 3D-printed human head (b) corresponding to the avatar seen in the virtual office. A rigid rod (c) articulated at its base for Dix–Hallpike maneuvers (not tested in this study) and supports the head by a spring (e) and 2 rubber bands (d) providing the haptic feedback for the passive head movements.

A training simulator for the examination of dizzy patients based on a VREM was developed. The system is composed of a virtual reality headset (HTC Vive^®^, HTC Corporation, Taoyuan, Taiwan), a computer, an articulated mannequin bearing a tracker on its vertex, and 2 controllers for calibration purposes (Fig. 4). The trainee wore the headset placing him/her in a virtual examination room in front of a virtual patient (Fig. 4). The avatar’s head position and dimensions corresponded to those of the articulated mannequin. The head was manufactured by 3D printing, mounted on a rigid rod with a spring and a rubber band to reproduce the movements of a flexible neck during the manipulations. It provided the haptic feedback during the examination (Fig. 4). The head was tracked by the simulation system via the tracker and the 2 detection cameras. Thus, the movements applied to the head were virtually reproduced by the avatar.

The software was developed on the Unity3D platform (Unity technologies, San Francisco, USA). The tracker position was sampled at 60 Hz. The delay between the dummy head movement and avatar’s movements was estimated at 58 ms. This delay includes: the transmission time between the movement of the tracker and its reception by the application (6 ms), the time for the application to calculate the position of the avatar (11 ms) and the time reaction of Unity3D (31 ms). The avatar movements were sampled at 120 Hz, for the calculation of the eye movements. The display (headset) refresh rate was 90 Hz. The system was run on a laptop computer (Intel^®^ CoreTMI7-8750H CPU at 2.2 GHz, and 16.0 GB RAM) with a graphic processor (GPU, NVIDIA GeForce RTX 2060) and 6 GB dedicated RAM.

**Figure 5:**
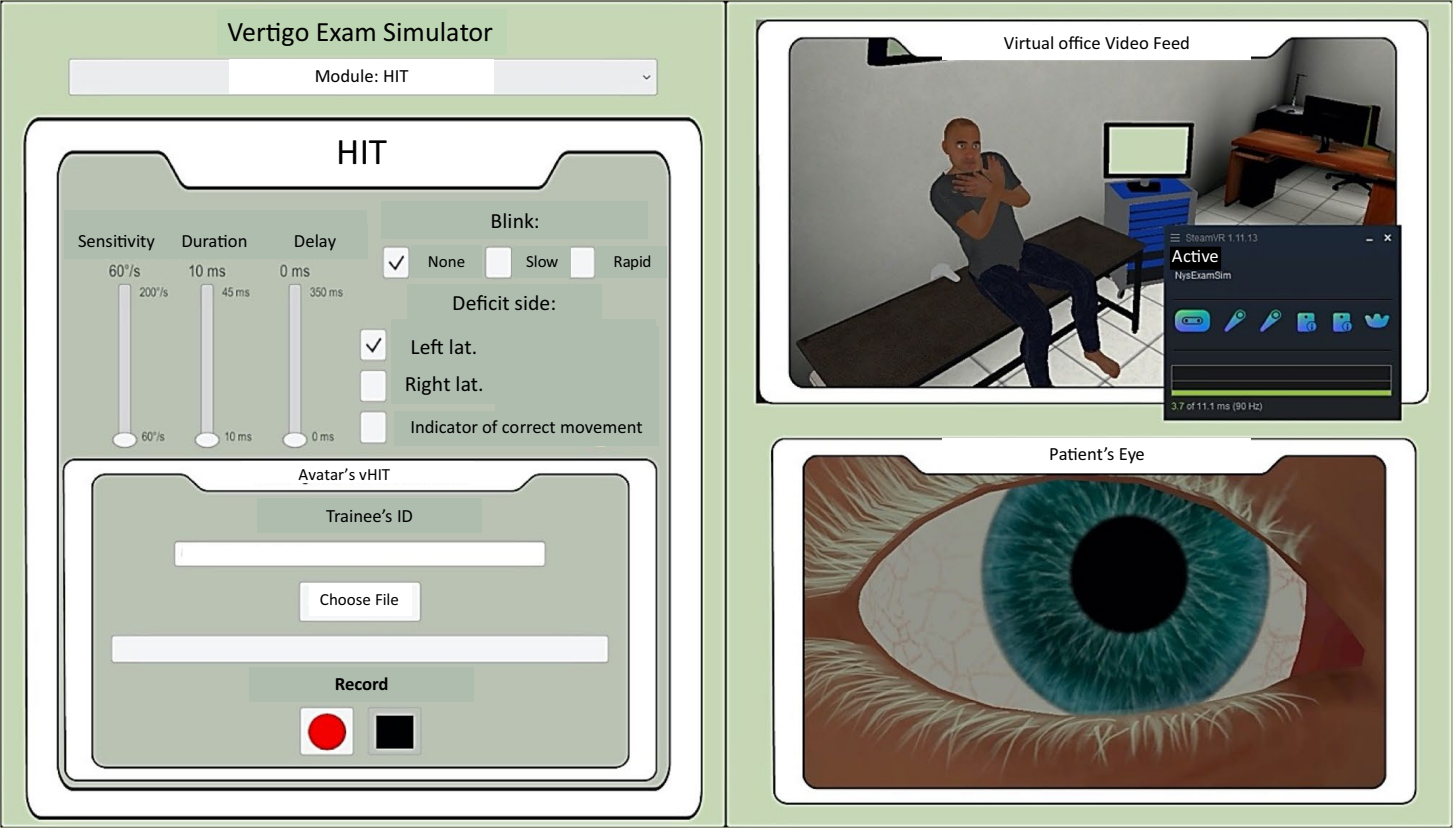
The control interface. The interface allows the trainer to change the parameters related to the head-impulse test scenario and to supervise the video feed from the virtual examination room and the patient’s right eye. It also allows to record the mannequins eye and head positions during the exercises.

The virtual patient stared at the trainee’s nose (estimated by the position of the headset) during the test. The control screen on the laptop (Fig. 5) allowed to supervise the virtual scene and to modify the following parameters: side of the horizontal canal deficit (right, left, both); speed threshold to trigger the saccade (range 60–200°/s), saccade duration (range 10–45 ms), saccade delay after the head movement (0–350 ms), random blinking (none, low at 0.03 Hz or high at 3 Hz).

**Figure 6:**
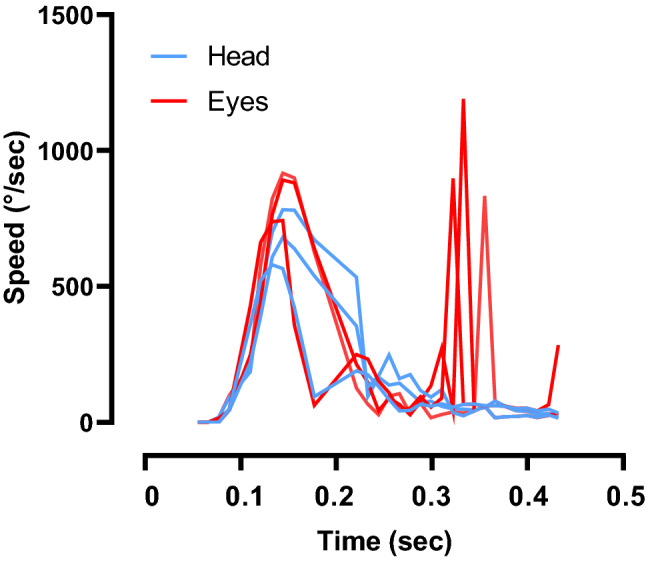
Recording of 3 virtual head impulse tests by the system. Eye and head positions were sampled at 120 Hz. In this example, the saccade (arrow) delay was set at 110 ms.

Head and eye movements were modelized according to vHIT data (Fig. 6). A virtual screen was also present in the virtual office above the avatar’s head to indicate a correct head impulse above the speed threshold by a green light. The screen was activated during the training phase for feedback and deactivated during the evaluation.

### Evaluation protocol

We conducted a prospective study from May to July 2020, including 35 novices and 7 expert volunteers to evaluate the detection of catch-up saccades during HIT and the effect of training in the virtual reality simulator. An informed consent was obtained from all subjects for both study participation and publication of identifying information/images. All methods were carried out in accordance with relevant guidelines and regulations in the Declaration of Helsinki. The protocol was reviewed and approved by the institutional ethical committee (CCP grand Est III). Trainees were medical residents, all with less than 1 year of experience in the field of vertigo. The experts were senior otolaryngologists specialized in otoneurology with more than 3 years of senior experience in the field. Subjects with visual, motor, or cognitive pathology which could interfere with the test or those with a known intolerance to simulation were excluded. At inclusion, the above inclusion and exclusion criteria were verified and data regarding age, sex, resident’s specialty, and seniority (semesters), hand laterality, prior knowledge of the HIT (yes/no), and prior practice of the test (yes/no) were collected.

For the novices, 2 trials were conducted, before and after a training session. They were subjected to a questionnaire regarding the test difficulty and the confidence on the observations after each series. Experts were submitted to only one trial, did not undergo training, and answered the same questionnaire.

The test was explained stereotypically as follows: "*This is a clinical test to determine the presence and the side of a vestibular deficit. It consists of asking the patient to stare at your nose. Then, you will take his head between your hands and apply a very rapid but small rotational movement in one direction and then in the other. To minimize the central compensation mechanisms, this movement should be unexpected and passive for the patient. Examine the patient's eyes carefully. If normal, the eyes will always remain fixed on your nose during the rotation. In case of a deficit, the eyes will leave their target, rotate with the head, and then return to the target (your nose) by a catch-up saccade. The presence of this saccade is the sign of a canal weakness on the side of the rotation and represents a positive test. You will perform this test several times in different conditions: the affected side changes and the difficulty may vary. For each condition, you can repeat the maneuver 5 times on each side before giving an answer among the following options: left deficit, right deficit, bilateral deficit or undetectable”.* Then, 2 videos of patients with a positive HIT were presented to the trainee. Subsequently, the volunteer was placed at the center of the simulator facing the dummy and wore the virtual reality headset.

The parameters were set for all tests as follows: head velocity trigger = 100°/s, saccade duration = 30 ms, blink = slow (random, 0.3 Hz). The saccade delays varied between 110 and 320 ms in 30 ms steps (8 increments) for each trial. Only lateral SCC deficits (left or right) were studied. Sixteen combinations of 8 delay increments and 2 deficit sides were presented in a computer-generated random order to avoid the order effect. For each condition, the subject was allowed 5 trials on each side (right and left) before answering (right, left, bilateral, undetectable).

Sensitivity (or true positive rate) was defined by to the ability to identify cases catch-up saccades on the correct side in case of a unilateral deficit. Specificity (true negative rate) corresponded to the ability identify cases without catch-up saccades and no deficit.

After the completion of the initial trial, the participant was subjected to 3 questions using a visual analogue scale (VAS):

- Difficulty in performing the maneuver (0: very easy to 10: very difficult)
- Difficulty in recognizing the saccades (0: very easy to 10: very difficult)
- Self-confidence in being able to provoke and detect saccades in a reproducible manner (0: not confident, 10: very confident)

The initial trial was followed by a training phase for novices. For the training, the session was carried out by the trainer on the dummy, to demonstrate the maneuver and the saccade using the computer screen. The trainee could then see the movement to be performed. He/she could also see, on the computer screen, the virtual patient's eye and the saccade which was indicated to him/her by the trainer. Then, the participant trained for 5 min to detect the saccade, knowing the side of the deficit. He/she could also check the validity of his movement via the virtual screen placed above the patient. A green light indicated a correct maneuver. The second trial for the novices was the same as the first series and included 16 randomized tests. Finally, the novice volunteers were again subjected to the 3 questions and provided their score by a VAS.

### Population

The novice group was selected among medical residents and was composed of 16 women and 19 men. Their mean age was 28.0 ± 2.33 years (range 25–32). Their average seniority was 5.5 ± 2.63 residency semesters (range 1–9). Two were left-handed and 33 were right-handed. Nine residents (26%) were familiar with the test and 8 (23%) had already practiced it on patients. The expert group was composed of 4 women and 3 men. Their mean age was 41.4 ± 11.83 (range 32–63). The average professional experience in the field of vertigo was 8.1 ± 6.77 years (range 3–20). In this group, there was one left-handed and 6 right-handed volunteers.

### Statistical analysis

Based on the hypothesis of an improvement in test sensitivity from 60 to 100% after training and considering a 40% dispersion, with an alpha error = 0.05, for a bilateral paired t-test, the number of subjects was estimated at 7 individuals in each subgroup (expert, novice)^41^. The number of subjects in the novice group was increased to 35 to insure the repeatability of the training effect.

Graphpad Prism software (v.6, Graphpad Inc., San Diego, CA, United States) was used for statistics. The quantitative descriptive variables were described as means and standard error of the mean (SEM), and the qualitative descriptive variables by frequencies and percentages. A Fisher test was used to compare true positives and false positives. The comparison of VAS scores (maneuver and detection) was performed using a repeated-measures ANOVA test followed by the Bonferroni test. The confidence VAS score was tested separately using a t-test. A p value < 0.05 was considered as significant.

Receiver operating characteristic curves were generated by Graphpad Prism software and area-under-the-curve (AUC) were compared via Pearson product-moment correlation of the AUCs according to Hanley and McNeil method^42^.

## Data Availability

The datasets used and/or analysed during the current study available from the corresponding author on reasonable request.
